# Effects of Zn-Enriched *Bifidobacterium longum* on the Growth and Reproduction of Rats

**DOI:** 10.3390/nu14040783

**Published:** 2022-02-13

**Authors:** Xinran Han, Fei Liu, Qiuxiang Zhang, Bingyong Mao, Xin Tang, Jie Huang, Renmei Guo, Jianxin Zhao, Hao Zhang, Shumao Cui, Wei Chen

**Affiliations:** 1 State Key Laboratory of Food Science and Technology, Jiangnan University, Wuxi 214122, China; 6190111026@stu.jiangnan.edu.cn (X.H.); 7200112081@stu.jiangnan.edu.cn (F.L.); zhangqx@jiangnan.edu.cn (Q.Z.); maobingyong@jiangnan.edu.cn (B.M.); xintang@jiangnan.edu.cn (X.T.); zhaojianxin@jiangnan.edu.cn (J.Z.); zhanghao61@jiangnan.edu.cn (H.Z.); chenwei66@jiangnan.edu.cn (W.C.); 2 School of Food Science and Technology, Jiangnan University, Wuxi 214122, China; 3 Suzhou Setech Biotechnology Co., Ltd., Suzhou 215000, China; jhuang@justug.com (J.H.); rmguo@justug.com (R.G.); 4 National Engineering Research Center for Functional Food, Jiangnan University, Wuxi 214122, China

**Keywords:** Zn, Zn-enriched *Bifidobacterium longum*, growth and reproduction development, gut microbiota

## Abstract

Zn is an essential trace element required for maintaining normal growth and development. Zn deficiency can cause growth retardation and reproductive system dysplasia, while Zn supplementation for treating Zn deficiency requires the use of high-quality Zn preparations. In this study, *Bifidobacterium longum* CCFM1195 was screened for its high Zn enrichment capacity, and the effects of different Zn supplementation regimens and doses on the growth and development of rats after Zn supplementation were investigated by supplementing Zn-deficient rat pups with different doses of various Zn supplements (ZnO, CCFM1195 + ZnO, and Zn-enriched CCFM1195). It was shown that the bioavailability of Zn was positively correlated with indicators of recovery after Zn supplementation, with Zn-enriched CCFM1195 having the best effect, followed by CCFM1195 + ZnO, while ZnO had the worst effect. Significant differences were also observed between the gut microbiota of control, model, and Zn-supplemented rats. Overall, administration of Zn-enriched CCFM1195 was more effective than the other approaches in restoring physical indicators of Zn deficiency after Zn supplementation, and this advantage was more significant at low-dose Zn supplementation.

## 1. Introduction

Zn is an essential trace element required for maintaining the normal growth and development of the human body. A human body contains 2–3 g of Zn, approximately 90% of which is found in muscles and bones [1]. Healthy adults consume approximately 10–15 mg of Zn daily through their diet, with a general absorption rate of 20–30%. Zn deficiency is a common nutritional deficiency worldwide, affecting about 31% of the global population, particularly in developing countries [2]. Notably, mild-to-moderate Zn deficiency is widespread [3]. Especially young children, as well as pregnant and postpartum women, are at a high risk of Zn deficiency [4]. Growth retardation and hypogonadism are known to result from a lack of Zn [5]. According to Fons et al. [6], 25 children with low serum Zn levels were characterized by substantially lower values of bone age delay, growth velocity in mm/month, and ratio between calculated and theoretical growth velocity for bone age. Zn supplementation during the right periods throughout development may also help to restore growth and development. After three months of Zn treatment, 50 pre-pubertal Egyptian children with low stature and Zn deficiency exhibited substantial increases in height standard deviation scores (SDS, *p* = 0.033) [7]. Furthermore, it has recently been identified that Zn is critical for male fertility, and that Zn shortage causes sperm abnormalities, as well as a decrease in serum testosterone concentration [1].

At present, Zn supplementation for Zn deficiency is mainly achieved by additional intake of Zn supplements, including inorganic Zn (e.g., ZnO, ZnSO_4_, and ZnCl_2_), simple organic Zn (e.g., Zn gluconate, Zn acetate, and Zn propionate), and organic Zn (e.g., amino acid-chelated Zn and protein-complexed Zn). However, the absorption efficiency varies significantly among the different Zn supplements [8,9]. Organic Zn is more easily absorbed by the human body than inorganic Zn, and the inorganic Zn is usually accompanied by side effects. Compared with the organic Zn, the simple organic Zn has certain side effects, such as irritation of the gastrointestinal tract. Organic Zn is mainly synthesized artificially, which is relatively safe, but its synthesis is complicated [10]. The enrichment of Zn by microorganisms has been extensively studied in recent decades [11]. Zn-enriched bacteria are produced during their growth on a Zn-containing medium [12] and the inorganic Zn can be converted into organic Zn, which is more easily absorbed and utilized. Probiotics are a kind of living microorganism, beneficial to the health of the host [13,14], and can be used in the field of Zn enrichment. In addition, the diversity and the stability of the intestinal microbiome will decrease significantly in the case of long-term Zn deficiency [15,16]. Zn-enriched probiotics can also regulate the intestinal microbiome [17] besides supplementing Zn. However, it remains unknown whether changes in the gut microbiota have an impact on the growth and reproduction of the host. As a new type of dietary Zn source, Zn-enriched probiotics have more advantages than other Zn supplements and are, thus, worth exploring deeply.

Given the presumed advantages of using Zn-enriched bacteria, we investigated the bioavailability of three different Zn supplements; that is, ZnO, *Bifidobacterium longum* + ZnO, and Zn-enriched *B. longum,* upon which Zn intake and the effects on growth and reproductive development of rats were examined. Using a Zn deficiency rat model, the effects of low, medium, and high doses of Zn supplementation on growth, reproductive development, Zn concentration in tissues, blood biochemistry, and the gut microbiota were assessed in rat pups. This study lays the foundations for the development of Zn supplements with high absorption efficiency and no side effects.

## 2. Materials and Methods

### 2.1. Screening of Strains with High Zn Enrichment

The 40 strains (Table A1) used in this study were all obtained from the Culture Collection Center of the Research Center of Food Biotechnology of Jiangnan University. The strains were isolated from fermented foods, such as fermented vegetables or fermented dairy products, and from feces of healthy babies, adults, and the elderly.

The 40 strains of *Lactobacillus plantarum*, *Lactobacillus reuteri*, *B. longum*, and *Bifidobacterium breve* were activated with mMRS medium (Table A2) for subsequent experiments. Activated strains were inoculated into liquid medium with a Zn^2+^ (ZnSO_4_) concentration of 200 mg/L at an initial inoculum proportion of 2%, cultured at 37 °C under anaerobic culture conditions for 12–18 h, and subsequently centrifuged at 8000× *g* for 15 min at 4 °C to obtain wet bacteria. The latter were rinsed twice with sterile ultrapure water to remove the Zn^2+^ remaining on the cell surface, followed by an additional centrifugation step to obtain the final wet bacteria. These were then resuspended in sterile ultrapure water and freeze-dried to obtain Zn-enriched bacterial powder. The Zn content of the bacteria was determined by flame atomic absorption spectrometry according to the method of Noriko et al. [18].

### 2.2. Morphological Analysis of Zn in Zn-Enriched Bacteria

The method described by Jia Yi [19] was used to analyze the morphological traits of bacteria + ZnO and Zn-enriched bacteria. Bacterial powder mixed with ZnO solution (using the same concentration as for the medium dose administered) and Zn-enriched bacterial powder were washed sequentially using water (pH = 6.5) and EDTA (0.01 mol/L, pH = 6.5). The Zn contents of the aqueous supernatants, EDTA supernatants, and digestions were determined.

### 2.3. Animal Experiment Design

All animal procedures were approved by the national “Regulations on the Management of Laboratory Animals” and the “Implementation Methods of Laboratory Animal Management in Jiangsu Province” committees. The following approval number from Jiangnan University’s laboratory animal welfare and ethics review (IACUC Issue No.) was assigned: JN.No20210930S0841108(361).

Fifty-five 3-week-old Sprague–Dawley rats were obtained from Charles River (Beijing, China) and maintained on a standard, non-purified rodent diet in solid plastic hanging cages under constant housing conditions (temperature, 24–26 °C; humidity, 40–60%), with a 12 h light and dark cycle. After one week of adaptation, all rats were randomly divided into control (*n* = 5), model (*n* = 5), ZnO (*n* = 15), bacteria + ZnO (*n* = 15), and Zn-enriched bacteria (*n* = 15) groups. Apart from the control group, all rats were fed a Zn-deficient diet (1 ppm, Trophic, Nantong, China) and provided with water ad libitum for a week of Zn deficiency modeling. The rats displayed symptoms, such as reduced feed intake, yellowish, dry, and dull hair, easy shedding, as well as perioral and interdigital dermatitis [20]. The control group was fed the control diet (35–38 ppm, Trophic, Nantong, China) and drank water ad libitum.

Rats from each experimental group were randomly assigned to one of the three Zn dose groups at the beginning of the fifth week, followed by daily gavage for two weeks using the following treatment regimens: (1) 0.2 mg Zn in 1 mL of 0.9% NaCl solution (low-Zn group, *n* = 15); (2) 0.7 mg Zn in 1 mL of 0.9% NaCl solution (medium-Zn group, *n* = 15); or (3) 2.1 mg Zn in 1 mL of 0.9% NaCl solution (high-Zn group, *n* = 15); rats from control and model groups were subjected to daily gavage for two weeks using 1 mL of a 0.9% NaCl solution (control, *n* = 5; model, *n* = 5). Medium Zn levels were chosen based on normal Zn intake levels (calculated based on a daily intake of 20 g feed). Low and high doses were set as one-third of and three times the medium level, respectively. All gavage solutions were freshly prepared on a daily basis prior to supplementation.

Throughout the gavage procedures, the weights were recorded every other day, and reaction, activity, food intake, mental status, and hair changes were all recorded regularly during the feeding period [20]. Feces were collected 24 h after the last gavage and the rats were fasted at night. At the end of the experiment, the rats underwent cardiac puncture under anesthesia. Zn was extracted from the blood, liver, testis, ileum, cecum, and colon for further analyses. Liquid nitrogen was used to flash-freeze liver, testis, ileum, cecum, and colon tissues, which were then stored at −80 °C. Blood samples were transferred to a procoagulant tube, and serum was collected after centrifugation. All samples were frozen and stored at −80 °C until further analysis. The bodies were temporarily stored in the freezer of the animal center and then handed over to a professional solid waste disposal company for unified disposal.

In addition, another experiment employing adult SD rats (IACUC Issue No.: JN.No20210930S0841108(361)) was designed to verify the absorption of ZnO, bacteria + ZnO, and Zn-enriched bacteria in vivo. Fifteen 8-week-old Sprague–Dawley rats were obtained from Charles River (Beijing, China), maintained under the conditions described above, and fed a Zn-deficient diet (1 ppm, Trophic, Nantong, China). After one week of adaptation, all rats were randomly divided into ZnO (*n* = 5), bacteria + ZnO (*n* = 5), and Zn-enriched bacteria groups (*n* = 5), and gavaged using the medium dose. Blood was taken from the inner canthus at 0.167, 0.5, 1, 2, 4, 6, 8, 12, and 24 h after gavage [21]. Approximately 0.4 mL of blood sample was withdrawn into a procoagulant tube and centrifuged at 3000× *g* for 15 min at 4 °C. The serum samples were frozen and kept at −80 °C until further analyses. At the end of the experiment, the rats were euthanized with CO_2_.

### 2.4. Blood Measurements

Serum samples were collected after centrifugation, divided, and stored at −80 °C for subsequent experiments.

#### 2.4.1. Zn Content

Serum samples were digested in HNO_3_ (16 mol/L) for more than 12 h, H_2_O_2_ was added, and digestions were performed at sub-boiling temperatures for 2 h [22]. After diluting the samples with deionized water, the Zn content was measured by flame atomic absorption spectrophotometer (Varian, Palo Alto, CA, USA).

#### 2.4.2. Alkaline Phosphatase (ALP) Activity

An automated biochemical analyzer (BC-5000, Mindray, Shenzhen, China) was used to determine the activity of ALP in the serum.

#### 2.4.3. Growth Hormone (GH) and Testosterone Levels

Rat GH enzyme-linked immunosorbent assay and Rat T ELISA Kits (Shanghai Enzyme-linked Biotechnology Co., Ltd., Shanghai, China) were used for determining the concentration of GH and testosterone in the serum according to the manufacturer’s instructions.

### 2.5. Tissue Zn Measurements

The liver, testis, ileum, cecum, colon, and feces were processed in the same way as the serum, with the exception that the sub-boiling digestion time was extended to 4 h [22]. After processing, the samples were evaluated in the same manner.

### 2.6. Microbial Profiling by 16S rRNA Sequencing

Before the final gavage, feces from each rat were collected and analyzed separately by 16S rRNA microbial profiling. DNA was extracted using the Fast DNA Spin Kit for Feces (MP Biomedicals, Santa Ana, CA, USA) according to the manufacturer’s standard technique. Polymerase chain reaction (PCR) was used to amplify the V3–V4 region of the 16S rRNA gene from each DNA sample using 314F (5′-CCTACGGGAGGCAGCAG-3′) and 806R (5′-GGACTACHVGGGTWTCTAAT-3′) primers. The following PCR reaction system is shown in Table A3 and the following PCR reaction conditions were employed: ① 95 °C for 5 min; ② 95 °C for 30 s, 52 °C for 30 s, 72 °C for 30 s, 35 cycles; ③ 72 °C for 7 min; ④ 12 °C for 5 min. The DNA Gel/PCR Purification Miniprep Kit (BW-DC3511-01, Biomiga, San Diego, CA, USA) was used to purify the PCR amplicons according to the manufacturer’s instructions. The purified DNA isolates were pre-processed on the genome of the extracted contents according to the on-machine requirements of the Illumina MiSeq platform and screened for high-quality sequencing data.

Raw data were processed using QIIME [23]. Sequences acquired were subjected to homologous alignment and cluster analysis. The categorization information on particular microbiome species was produced by comparing the sequencing information obtained from OTU cluster analysis with the Silva database (SILVA128) [24], after which the composition and percentage of the bacteria were assessed. At the taxonomic level, the makeup of the gut microbiota in each sample was examined at phylum, class, order, family, and genus levels. To quantify microbial abundance and diversity, the alpha diversity of the microbial species present in the samples was evaluated. Meanwhile, Chao1 indices were generated to examine species diversity. The variations in species diversity within the samples were examined using beta diversity, with principal coordinates analysis (PCoA) being utilized to compute individual species differences across samples. The abundance differences of marker species across groups were analyzed using Linear discriminant analysis Effect Size (LEfSe) analysis.

### 2.7. Statistical Analysis

The experimental data were statistically analyzed by SPSS 22.0 (IBM Corporation, Chicago, IL, USA). All statistical analyses were performed using OriginPro 2017 (OriginLab Corporation, Northampton, MA, USA). The results are presented as mean ± SEM. Each experiment was repeated at least three times in parallel and analyzed by one-way ANOVA. When a significant impact was confirmed by ANOVA, Duncan’s test was used to analyze differences across groups. Statistical significance was set at a *p* < 0.05. To approach a normal distribution, data on relative microbial abundances were arcsine converted.

## 3. Results

### 3.1. Zn Enrichment Ability of Different Bacterial Strains

**Figure 1:**
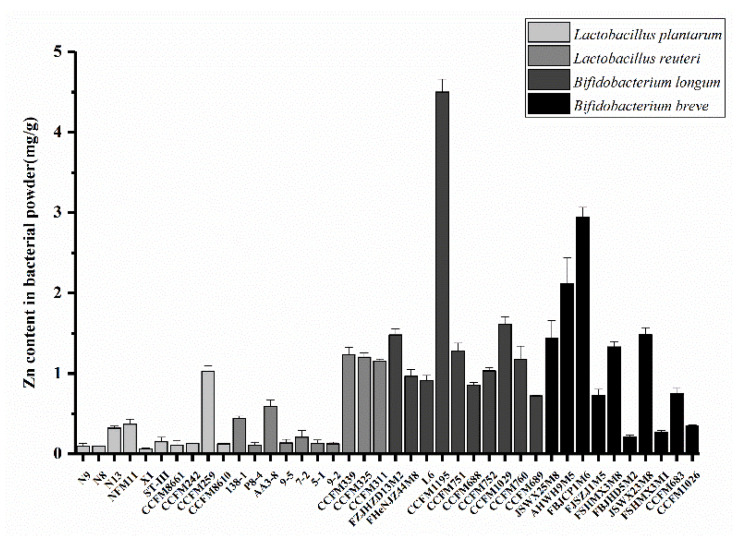
Ability of different strains to enrich Zn in the medium with an initial Zn concentration of 200 mg/L. Data are presented as mean ± SEM (*n* = 5).

According to the method mentioned above, the Zn^2+^ enrichment ability of different strains was determined, and the Zn content in the freeze-dried bacterial powder was used to characterize the Zn^2+^ enrichment ability. Strains with high Zn enrichment ability were screened. Figure 1 shows the enrichment capacity of different strains of Zn^2+^ in a Zn-containing medium. The initial concentration of Zn^2+^ in the medium was 200 mg/L. The results indicated that different strains showed great differences in Zn enrichment ability, with the enrichment amount ranging from 0.06–4.50 mg/g. Furthermore, the Zn enrichment ability of *Bifidobacteria* was generally better than that of *Lactobacilli*. In this experiment, the *B. longum* CCFM1195 strain displayed the highest Zn content and was, therefore, selected as the strain to be used in subsequent experiments. The number of viable bacteria and Zn content in *B. longum* CCFM1195 lyophilized bacterial powder (including lyophilized protective agent) are shown in Table 1. The number of viable *B. longum* CCFM1195 was 3.8 × 10^8^ cfu/g, and it was 1 × 10^9^ cfu/g when Zn-enriched and the Zn content was 1.7 mg/g.

### 3.2. Morphological Analysis of Zn in B. longum CCFM1195 + ZnO and Zn-Enriched B. longum CCFM1195

The Zn contents in CCFM1195 + ZnO and Zn-enriched CCFM1195 powders were analyzed, including inorganic Zn on the cell surface, organic Zn bound to the cell wall, and intracellular Zn. The H_2_O supernatant was used to measure the content of water-soluble Zn (inorganic Zn) on the cell surface. The EDTA supernatant was used to detect Zn contents of cell wall polysaccharides and protein complexes. Digestions were performed to test the content of organic macromolecules or small molecules bound to Zn in cells. The results of these analyses are shown in Table 2 and Table 3. After the CCFM1195 powder was fully mixed with the ZnO solution, the organic Zn content was determined to be 45.49%. Most of the organic Zn was bound to the cell wall. While the organic Zn content of Zn-enriched CCFM1195 was 96.47%, most of it was present inside the cells.

### 3.3. Apparent Indicators of Growth and Reproduction

**Figure 2:**
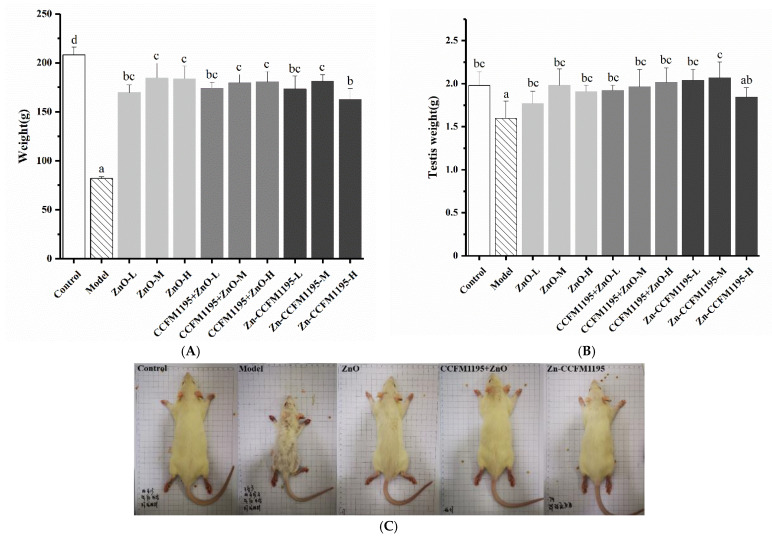
Effects of Zn supplementation on apparent characteristics of growth and reproduction. (**A**) Rat weight on the last day of gavage. (**B**) Testis weight of rats. Data are presented as mean ± SEM (*n* = 5). Different letters indicate significant differences (*p* < 0.05) based on Duncan’s test. (**C**) Photos of rats. From left to right: one rat each from control, model, ZnO, CCFM1195 + ZnO, and Zn-enriched CCFM1195 groups. Medium doses were employed in the case of all Zn treatment groups included.

The growth and reproductive development of pups in each group are shown in Figure 2. Figure 2A,B indicate the weights and testis weights of the rats, respectively. It was revealed that dietary Zn deficiency could seriously affect the growth (Figure 2A) and reproductive development (Figure 2B) of the pups during the examined growth period. The differences in body and testicular weights across the Zn supplementation groups were not statistically significant, although they were all considerably greater than that of the model group, and somewhat lower than that of the control group. This suggested that Zn supplementation after Zn deficiency could effectively improve the growth and reproductive dysplasia of rat pups, while Zn supplementation may be necessary for longer periods to fully restore normal growth and reproductive development [7]. Studies have reported that Zn renewal in a human body takes approximately 12.5 to 300 days. The liver, which is considered the tissue with the fastest Zn metabolism, also needs more than 30 h for turnover [25].

Figure 2C features pictures of rats randomly selected from control, model, and medium-dose groups, treated with different Zn supplements. The rats from the model group were much smaller than those in the other groups, as seen in Figure 2C. Alopecia was observed throughout the whole body of the Zn-deficient rat, which was accompanied by dermatitis between the toes. This was consistent with previous reports on the phenomena caused by Zn deficiency in rats [26,27]. Less pronounced hair loss was also observed on the back of the rat in the ZnO group. In conformity with the weights of the rats (Figure 2A), the pups in the Zn treatment group were slightly smaller in body size than the control rats.

### 3.4. Zn Content in Tissue

**Figure 3:**
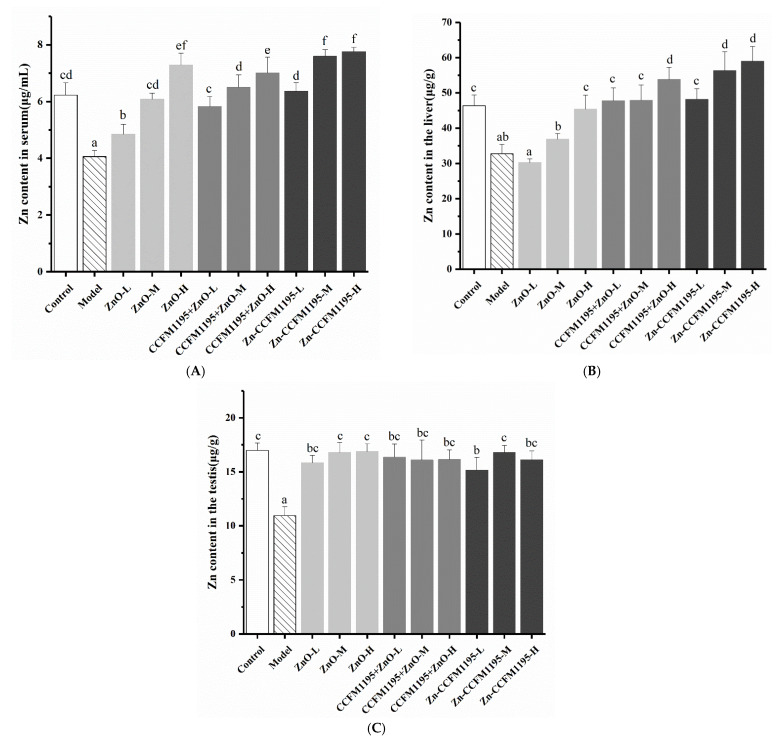
Effects of Zn supplementation on the Zn content in different tissues. (**A**) Zn content in serum, an indicator of Zn absorption. (**B**) Zn content in the liver, an indicator of Zn utilization. (**C**) Zn levels in the testes, indicative of the use of Zn by the testes. Data are presented as mean ± SEM (*n* = 5). Different letters indicate significant differences (*p* < 0.05) based on Duncan’s test.

Although there was no significant difference among the different Zn treatment groups, with respect to apparent indicators, further studies were conducted in order to find the mechanisms involved. The Zn content detected in the serum, liver, and testis is shown in Figure 3A–C. Dietary Zn deficiency could significantly lower the Zn levels in the organs analyzed. Serum Zn reflects the absorption of different Zn supplements by the body, while hepatic Zn reflects its utilization [28]. The testicular Zn content was indicative of the accumulation of different Zn sources in the testis. Serum Zn levels and the Zn deposits in the liver are used as markers of Zn bioavailability [29,30,31]. It was identified from Figure 3 that the amount of Zn supplemented was proportional to its absorption and utilization by the body. However, absorption and utilization rates were different in response to various Zn sources. Zn in the form of Zn-enriched CCFM1195 was associated with much greater absorption efficiency than Zn in the form of ZnO, whereas treatment with CCFM1195 + ZnO resulted in intermediate absorption efficiencies (Figure 3A). At a low dose, there were profound differences in the degree of absorption of the different Zn sources in the rats. A similar tendency also existed in the case of medium and high doses but was less significant. Moreover, the extent of Zn utilization followed the same trend as that of Zn absorption (Figure 3B). *Bifidobacterium longum* CCFM1195 could enhance the absorption and utilization rates of ZnO, regardless of the dosage used. Zn supplementation significantly affected the recovery of Zn levels in the testes of Zn-deficient rats (Figure 3C). There was no significant difference in the deposits of different Zn sources in the testes, but they were all significantly higher than in the model group. Therefore, we speculated that Zn absorbed by the body is preferably supplied to the reproductive system, which needs more Zn after being utilized [4], to restore and maintain the normal function of the testes.

### 3.5. Activity Differences of Zn-Related Enzymes

**Figure 4:**
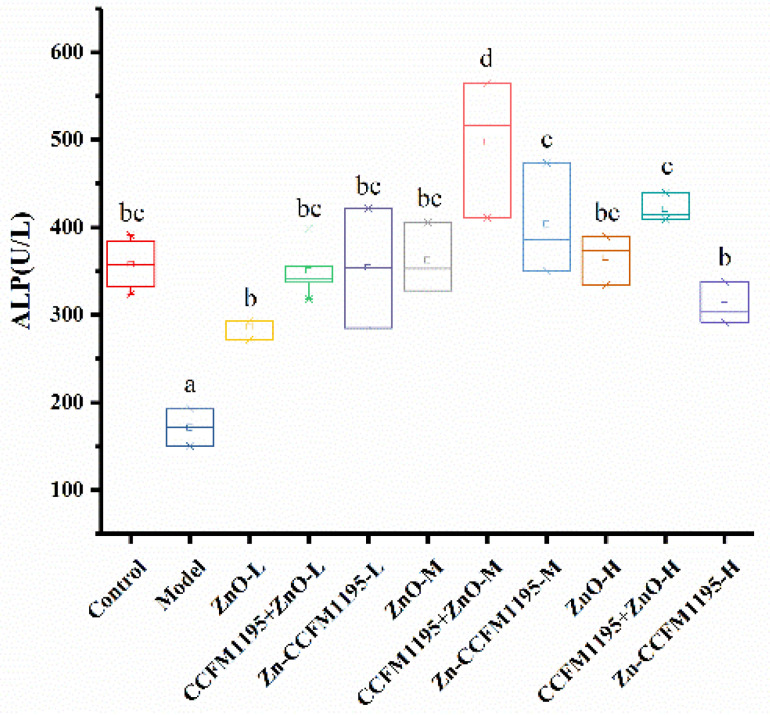
Activities of alkaline phosphatase (ALP) in serum. Different letters indicate significant differences (*p* < 0.05) based on Duncan’s test.

The activity of ALP is closely linked to Zn levels; many scholars believe that ALP is a simple and feasible indicator to accurately reflect the status of zinc metabolism in the body [32]. Figure 4 shows the activity of ALP in serum. It could be observed that additional Zn supplementation could quickly restore the activity of Zn-related enzymes in rats by comparing the Zn supplementation groups with the control group. The Zn-deficient group displayed significantly lower ALP enzyme activity than the control and Zn-supplemented groups, indicating that Zn deficiency had a significant effect on Zn-dependent enzymatic systems. In terms of the ability to restore the activities of the ALP enzyme, Zn-enriched CCFM1195 elicited the best results, while ZnO performed worst in the low Zn groups. Bifidobacterium longum CCFM1195 could improve the recovery ability of ZnO with respect to the enzyme. In the medium and high Zn groups, compared with the CCFM1195 + ZnO group, the recovery potential of Zn-enriched CCFM1195 with respect to ALP enzyme activity was decreased, suggesting that Zn-enriched CCFM1195 was easily absorbed and utilized by the body, resulting in excessive Zn levels that had a negative impact on the enzymatic activity.

### 3.6. GH and Testosterone in Serum

**Figure 5:**
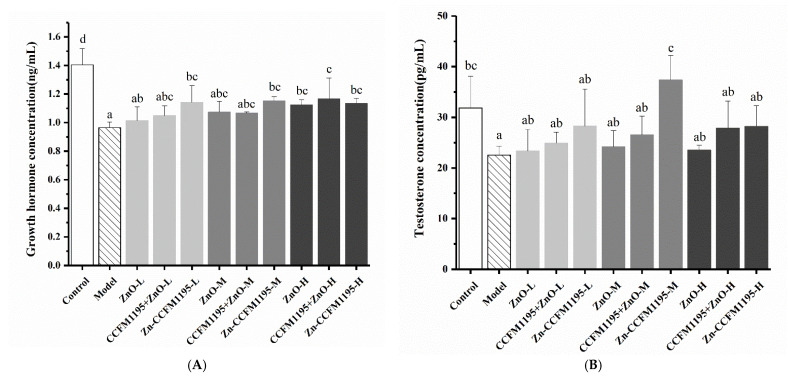
Effects of Zn supplementation on the growth hormone and testosterone concentrations in serum. (**A**) Growth hormone concentration. (**B**) Testosterone concentration. Data are presented as mean ± SEM (*n* = 5). Different letters indicate significant differences (*p* < 0.05) based on Duncan’s test.

To estimate the influence of different Zn levels on body functions, GH and testosterone serum concentrations were measured (Figure 5). In contrast to the control group, Zn deficiency could significantly reduce the concentration of GH and testosterone in the serum. Compared with the model group, only Zn-enriched CCFM1195 could increase GH levels significantly at low and medium doses, while all treatments elicited significant differences at high doses. Unfortunately, although additional Zn supplementation improved the testosterone concentration as a function of the dose administered, the difference was not significant compared to the Zn-deficient group. Therefore, it is conjectured that Zn supplementation may take longer to affect GH concentrations or that Zn may act on targets downstream of testosterone to regulate reproductive development.

### 3.7. Zn Content in the Intestinal Tract

**Figure 6:**
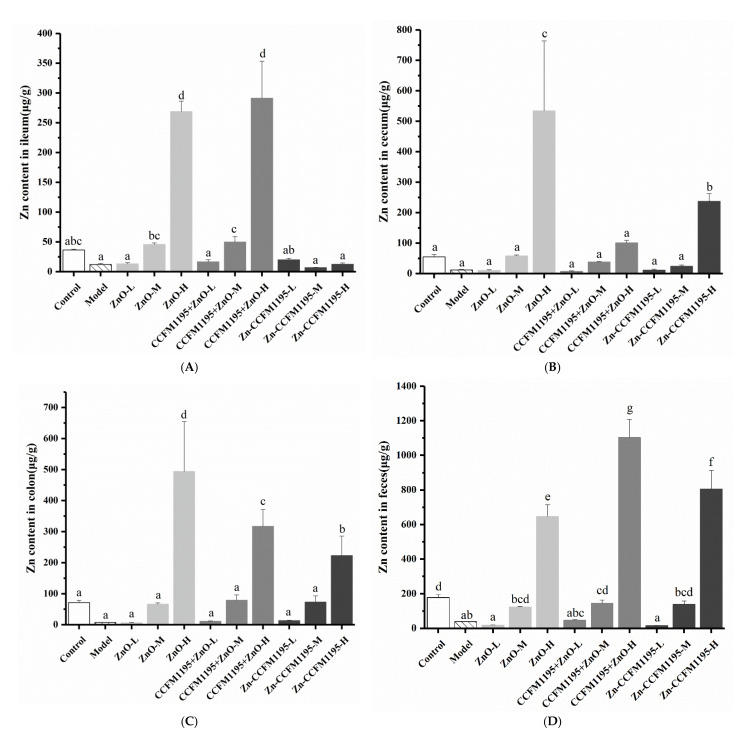
Effects of Zn supplementation on the Zn content in the intestinal tract. (**A**–**C**) Zn contents in the ileum, cecum, and colon, respectively, representing the Zn absorbed. (**D**) Zn content in feces, representing the Zn excreted in the stool. Data are presented as mean ± SEM (*n* = 5). Different letters indicate significant differences (*p* < 0.05) based on Duncan’s test.

To evaluate the causes of these differences, the Zn content of different intestinal segments was determined, which implied a difference in the absorption and utilization of different Zn supplements at different doses. The Zn content in the ileum, cecum, and colon reflected the accumulation of different Zn sources in each intestinal segment, which is shown in Figure 6. It was revealed that the amount of Zn supplemented was proportional to the Zn accumulated in each intestinal segment. This was also dependent on the type of the Zn supplement. Based on the Zn content detected in each intestinal segment, it could be concluded that the accumulation of Zn was greatest in the cecum, less pronounced in the colon, and least in the ileum. This phenomenon was in line with the accumulation of trace elements in various intestinal segments after their intake [33]. The accumulation of Zn-enriched CCFM1195 in the ileum was smallest, followed by ZnO (Figure 6A). Compared with the ZnO groups, *B. longum* CCFM1195 could increase the accumulation of ZnO in the ileum. There was no significant difference in the accumulation of each Zn supplement in the ileum at a low dose. In both the medium and high Zn groups, the accumulation of Zn in the ileum was highest for CCFM1195 + ZnO, but less for ZnO. In the cecum, the accumulation of ZnO was highest, followed by Zn-enriched CCFM1195 (Figure 6B). *Bifidobacterium longum* CCFM1195 reduced the accumulation of ZnO in the cecum, when compared to the ZnO groups. There was neither a significant difference in Zn accumulation among the three different Zn supplements at a low dose nor at a medium dose. However, the accumulation of ZnO was highest, followed by Zn-enriched CCFM1195, whereas the accumulation of CCFM1195 + ZnO was lowest at a high dose. The accumulation of Zn in the colon, in the form of CCFM1195 + ZnO, was greater than in the case of Zn-enriched CCFM1195, but smaller than for ZnO (Figure 6C). No significant difference in the accumulation of various Zn supplements in the colon was observed in the low Zn groups. At a high dose of Zn, the accumulation in the colon was greater after ZnO than after Zn-enriched CCFM1195 treatment, with accumulation after CCFM1195 + ZnO being intermediate.

Fecal Zn levels after 2 weeks of gavage are shown in Figure 6D. Fecal samples were collected 24 h after the last gavage. According to the Zn content detected in the feces, it was concluded that although Zn excretion was in proportion to Zn intake, the fecal Zn content of rats supplemented with different Zn sources was different. At low-dose Zn supplementation, the Zn content in the feces of rats treated with various Zn supplements was not significantly different, and the same phenomenon was observed at medium doses. At high doses, however, a profound difference was observed among the fecal Zn content of rats supplemented with different Zn sources. The fecal Zn content in the ZnO and CCFM1195 + ZnO groups was the smallest and highest, respectively. Fecal excretion of Zn by the Zn-enriched CCFM1195 group was, accordingly, intermediate. It was speculated that inorganic Zn, which is a small molecule, could not stay in the intestine for an extended period of time. As the samples were collected at 24 h after gavage, inorganic Zn had probably been excreted already, while viable bacteria prolonged the residence of ZnO in the intestinal tract.

### 3.8. Pharmacokinetic Data Analysis

**Figure 7:**
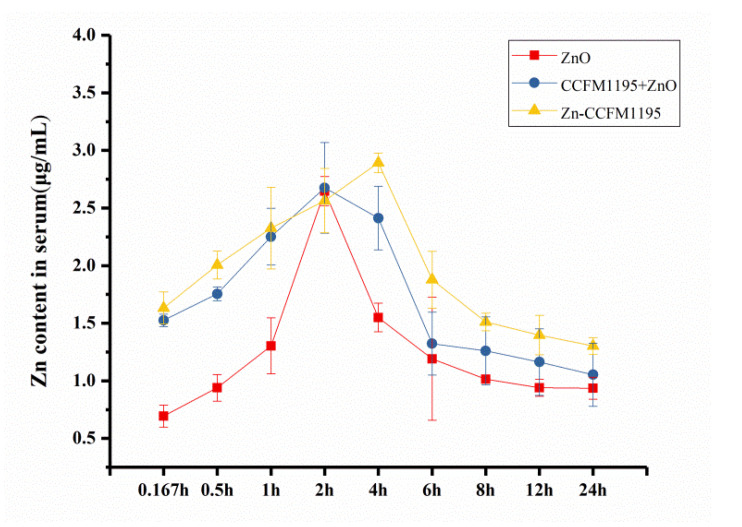
Pharmacokinetic analysis of adult SD rats. Data are presented as mean ± SEM (*n* = 5).

The absorption of different Zn supplements was evaluated in vivo by analyzing the serum Zn content at 0.167, 0.5, 1, 2, 4, 6, 8, 12, and 24 h after gavage. Figure 7 shows the change curve of the serum Zn content. It can be seen that the serum Zn content of the ZnO and CCFM1195 + ZnO groups peaked at 2 h after gavage, while the serum Zn content of the Zn-enriched CCFM1195 group peaked at 4 h after intragastric administration. Among the different groups, the peak serum Zn of Zn-enriched CCFM1195 was the highest. The serum Zn content in the ZnO group decreased rapidly after reaching the peak value, and the residual serum Zn content in the body was the lowest. Both CCFM1195 and Zn-enriched CCFM1195 increased the serum Zn content and prolonged the retention time of Zn in the body compared with the ZnO group.

### 3.9. Gut Microbiota

**Figure 8:**
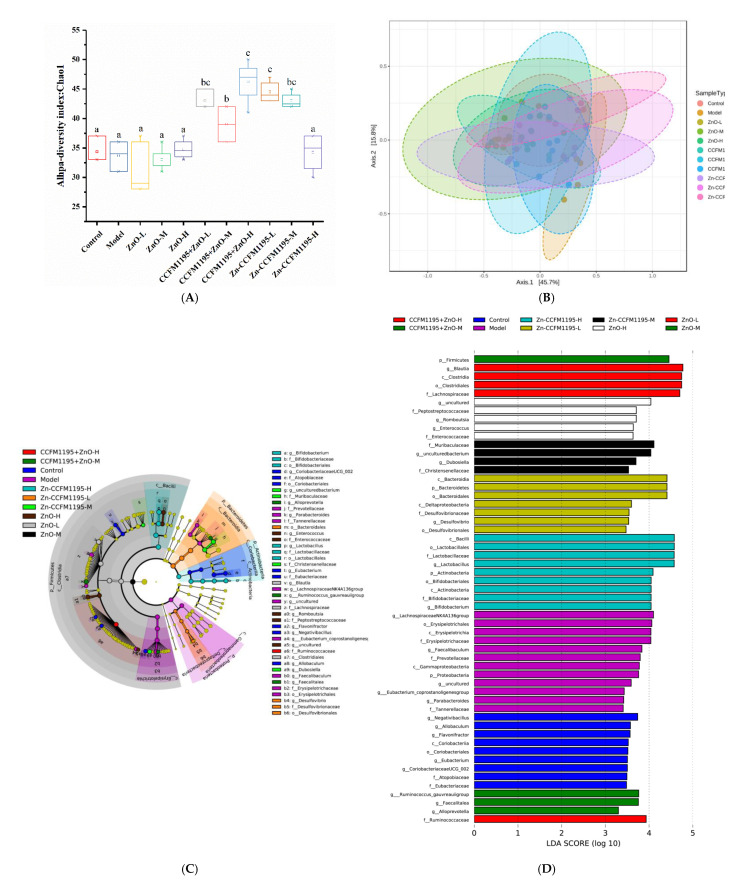

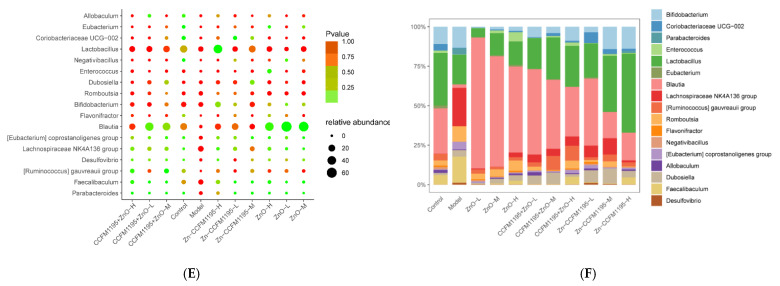
Alpha diversity, beta diversity, and LEfSe analysis. (**A**) Alpha diversity of bacterial communities in different treatment groups. The total number of microbial species in samples is represented by Chao1 indices, observed species, and Good’s coverage. Different letters indicate significant differences (*p* < 0.05) based on Duncan’s test. (**B**) Principal coordinates analysis (PCoA) results of each sample. (**C**) Linear Discriminant Analysis Effect Size (LEfSe) analysis identified the most differentially abundant taxon between each group. The evolutionary branch diagram depicts the taxonomic phylum, class, order, family, genus, and species from center to periphery. At each categorization level, each node represents a species. Species with no notable differences are indicated by yellow nodes. (**D**) Species with an LDA score > 3. (**E**) P values of relative abundances of the microbiota markers at the genus level in the different groups. The p values were calculated by comparison with the model group. (**F**) Composition of the sample of intestinal bacteria at discrepant genus, expressed in relative abundance.

Microbial 16S rRNA profiling of feces from rats after 2 weeks of gavage was undertaken to explore the interaction between dietary Zn, the intestinal microbiota, and the host. Alpha and beta diversities, as well as LEfSe analyses, were used to evaluate the species present, their quantities, and thus, the composition of the gut microbiota. The alpha diversity of the intestinal microbes in each group was analyzed and was used to calculate the microbial abundance and diversity of the samples, while the species richness was estimated using Chao1 indices (Figure 8A). There was no significant difference in intestinal diversity between control, model, and ZnO groups. However, regardless of whether CCFM1195 + ZnO or Zn-enriched CCFM1195 were used, an increase in diversity in the gut microbiota was observed, which was statistically significant (*p* < 0.05), indicating that *B. longum* CCFM1195 played an important role in shaping the gut microbiota. Interestingly, in the Zn-enriched CCFM1195-H group, the diversity was reduced.

Beta diversity analysis is a technique for analyzing the variations in species’ diversity of samples, as a tool for studying the similarities of the overall community composition in samples. Individual differences among samples and sample duplications within groups were calculated using PCoA (Figure 8B). Samples from the model group, which had low species diversity and abundance, and the CCFM1195 + ZnO-H group, which displayed high species diversity and abundance, were separated from samples from the other groups by a significant distance, although there was no discernible difference. The close proximity of the other groups showed that their microbial compositions were more comparable.

LEfSe analyses were used to distinguish individual species across groups, allowing for the presentation of species categorization information, as well as abundance differences between groups. At the taxonomic level, the graph of evolutionary branches represents the phylum, class, order, family, genus, and species from center to periphery. The quantity of the species was positively associated with the size of each node that represents a species at each categorization level. Species with no substantial differences are indicated by yellow nodes (Figure 8C). Biological indicators for each category were species having an LDA score greater than three (Figure 8D). The microbiota markers in the control group were *Negativibacillus*, *Allobaculum*, *Flavonifractor*, *Coriobacteriia*, *Coriobacteriales*, *Eubacterium*, *Coriobacteriaceae* UCG_002, *Atopobiaceae*, and *Eubacteriaceae*. The microbiota markers in the model group were *Lachnospiraceae* NK4A136 group, *Erysipelotrichales*, *Erysipelotrichaceae*, *Faecalibaculum*, *Prevotellaceae*, *Gammaproteobacteria*, *Proteobacteria*, *Eubacterium coprostanoligenes* group, *Parabacteroides*, and *Tannerellaceae*. The microbiota markers of the ZnO-L group were *Blautia*, *Clostridia*, *Clostridiales*, and *Lachnospiraceae*. The microbiota marker in the ZnO-M group was *Firmicutes*. The microbiota markers in the ZnO-H group were *Peptostreptococcaceae*, *Romboutsia*, *Enterococcus,* and *Enterococcaceae*. The microbiota markers in the CCFM1195 + ZnO-M group were *Ruminococcus gauvreauii* group, *Faecalitalea*, and *Alloprevotella*. The microbiota marker in the CCFM1195 + ZnO-H group was *Ruminococcaceae*. The microbiota markers in the Zn-CCFM1195-L group were *Bacteroidia*, *Bacteroidetes*, *Bacteroidales*, *Deltaproteobacteria*, *Desulfovibrionaceae*, *Desulfovibrio*, and *Desulfovibrionales*. The microbiota markers of Zn-CCFM1195-M were *Muribaculaceae*, *Dubosiella*, and *Christensenellaceae*. The microbiota markers in the Zn-CCFM1195-H group were *Bacilli*, *Lactobacillales*, *Lactobacillaceae*, *Lactobacillus*, *Actinobacteria*, *Bifidobacteriales*, *Bifidobacteriaceae*, and *Bifidobacterium*.

According to the LEfSe analysis, the microbiota markers at the genus level were selected for further studies. The significant differences and relative abundances of these discrepant genera were investigated (Figure 8E,F). The intestinal microbiota compositions of rats in the model and intervention groups were substantially different from the control group, according to an analysis of bacterial OTU abundance at the genus level. There was a significant increase in the *Lachnospiraceae* NK4A136 group, and a reduction in the *Ruminococcus gauvreauii* group, *Flavonifractor*, and *Negativibacillus* in Zn-deficient rats. In the Zn intervention groups, *Blautia* decreased with Zn intake, while *Lactobacillus* increased with Zn intake, regardless of the Zn supplements used.

## 4. Discussion

Zn supplementation could improve the growth and reproductive development of Zn-deficient rat pups [34,35], despite no significant variations in body weight and testicular weight between intervention groups. We still considered them crucial, particularly due to the importance of internal factors. In contrast, body and testicular weights in the high dose group of Zn-enriched CCFM1195 were slightly decreased. We speculated that the Zn levels in the body were higher due to higher bioavailability, which increased the burden of metabolizing this micronutrient. While the liver is the main organ involved in Zn metabolism, the small intestine, liver, and pancreas maintain Zn homeostasis [36]. It has been documented that excessive Zn will reduce the weight of the pancreas [37]. In addition, an excess of Zn will compete with other micronutrients [38], affecting the absorption of copper, iron, calcium, and other elements, which will also have adverse effects on the body.

The inner connection caused by different Zn supplements at different doses will inevitably lead to different levels of Zn in the serum and organs. Zn absorbed by the intestine first enters the blood, combines with albumin in the blood (above 94%), and is then transported to the liver (about 67–80% of total Zn absorbed) [39]. The utilization ratio of the Zn absorbed, as evaluated by serum Zn levels and Zn deposits in the liver, was highest for Zn-enriched CCFM1195, less for CCFM1195 + ZnO, and least for ZnO. The trend was even more pronounced at low Zn supplementation. This result also verified our previous speculation on the Zn absorption efficiency associated with the three Zn supplements. With respect to a normal diet, many factors affect the bioavailability of Zn, such as phytic acid, high dietary fiber, and polyphenols, which form insoluble complexes with Zn [40], reducing the bioavailability of exogenous Zn and decreasing the absorption of Zn in the small intestine. In this case, most of the exogenous Zn will be transported to the colon to compensate for the impaired absorption of Zn in the proximal region [41]. In addition, other metal ions, such as Ca^2+^ or Fe^2+^, compete with Zn^2+^ for intestinal absorption [38]. Zn-enriched probiotics chelate Zn with suitable complexing strength. Because trace elements are protected by the ligand after entering the digestive tract, this approach can help avoid the influence of physical and chemical factors that are not conducive to metal absorption in the gastrointestinal tract, reduce the antagonism among different metal ions, and the negative effects of phytate on the absorption of Zn in the small intestine [8,42], thereby increasing the absorption rate. Short-chain fatty acids (SCFAs) are produced by bacterial fermentation and serve as the main metabolic substrate of colon cells [43]. As such, SCFAs can increase dietary Zn absorption by decreasing the pH in the intestinal lumen [44], thus, increasing the solubility of Zn, or by stimulating the proliferation of intestinal epithelial cells to increase the total absorption area of the intestine [45]. Furthermore, studies have shown in recent years that some probiotics (mainly *Lactobacillus* and *Bifidobacterium*) can degrade oxalate in the intestine [46,47,48,49,50]. Together, these studies may help explain why the bioavailability of Zn-enriched CCFM1195 and CCFM1195 + ZnO was higher than that of ZnO alone. However, why the bioavailability of Zn-enriched CCFM1195 was in turn higher than that of CCFM1195 + ZnO is not yet known; it may be dependent on the ratio of organic to inorganic Zn, which must be confirmed by further studies. At present, we have proven that the proportion of organic Zn in Zn-enriched CCFM1195 is much higher than that in CCFM1195 + ZnO and that their morphologies differ. The organic Zn of Zn-enriched CCFM1195 comprises mainly Zn bound to intracellular molecules, whereas that of CCFM1195 mixed with ZnO comprises mainly Zn bound to the cell wall.

The different Zn levels in the body, in turn, generated activity differences of Zn-dependent enzymes and changes in hormone levels. Ultimately, growth and reproductive development will be affected. Different Zn supplements had different abilities to restore ALP enzyme activity. Our results indicated that Zn-enriched CCFM1195 had the strongest ability to restore enzyme activity, followed by CCFM1195 + ZnO, while ZnO was the worst, which was in line with previously obtained results. In terms of the GH and testosterone concentrations in the serum, our results showed that the ability of Zn-enriched CCFM1195 to increase the GH concentration was most significant. Additional Zn supplementation improved the testosterone concentration as a function of the dose administered, but no significant difference in testosterone concentration was observed compared to the Zn-deficient group. However, additional Zn supplementation resulted in significant differences in testis weight and testicular Zn content, indicating that Zn may act on targets downstream of testosterone to regulate reproductive development.

The cause of all these differences may have risen from the difference in absorption and utilization of the different Zn supplements at different doses by the body. The Zn content in the ileum, cecum, colon, and feces was measured. Zn is mainly absorbed in the small intestine, with very little absorption in the stomach and an auxiliary effect within the large intestine [51]. The accumulation of Zn in each intestinal segment can be considered indicative of the state of Zn to be absorbed. In general, ZnO led to the highest accumulation rates in the three intestinal segments, followed by CCFM1195 + ZnO, while Zn-enriched CCFM1195 was associated with the lowest accumulation rates. Unabsorbed dietary Zn (exogenous Zn) and endogenous Zn produced from pancreatic and biliary secretions, gastroduodenal secretions, transepithelial flow from enterocytes or other intestinal cell types, and mucosal cell sloughing, were all excreted in the feces [36,52]. There is a Zn homeostasis regulation mechanism in animals, and Zn absorption and endogenous secretion are the main ways of Zn homeostasis regulation [53]. The gastrointestinal system, particularly the small intestine, liver, and pancreas, is thought to play a major role in maintaining Zn homeostasis [52]. The maintenance of Zn metabolism in the body mainly depends on the regulation of intestinal excretion. When the body takes in too much Zn, the amount of Zn secreted into the small intestine by the liver and pancreas increases, thus, increasing endogenous Zn exclusion to achieve a regulation of Zn supply. Zn is released from the body into the colon without being absorbed, resulting in endogenous Zn loss via the feces. The major source of endogenous Zn re-excretion into the intestinal lumen is pancreatic production (which is six times higher than secretion through bile) [54]. Researchers have reported that an enteropancreatic circulation exists in humans. Much of this Zn must eventually be resorbed to avoid a negative Zn balance. According to the Zn content in feces collected after 24 h of gavage, ZnO could not stay in the intestinal tract for a long time and was excreted soon after being eaten, resulting in the lowest fecal Zn content in the ZnO groups. Zn-enriched CCFM1195 could stay in the gut for a long time. Compared with the ZnO groups, *B. longum* CCFM1195 could increase the residence time of ZnO in the intestine, which may have increased the total amount of ZnO absorbed and promoted the absorption process. Therefore, we speculated that Zn was more efficiently absorbed when administered as Zn-enriched CCFM1195 than when given as ZnO. CCFM1195 + ZnO could be placed between the other two Zn supplements in terms of absorption efficiency.

Pharmacokinetic data analyses confirmed our previous speculation on the absorption of ZnO, CCFM1195 + ZnO, and Zn-enriched CCFM1195. The absorption and metabolism pathways of inorganic and organic Zn are different. Inorganic Zn was not readily absorbed, and only when transformed into organic Zn could be absorbed, transported, and utilized by the body. Organic Zn existed stably in the digestive tract and was absorbed by the brush-like border of the small intestinal villi, in the form of amino acids or peptides. It did not form complexes with cellulose and phytic acid, which are difficult to be absorbed and utilized [42]. Moreover, antagonistic effects between elements were also avoided. ZnO was quickly excreted through absorption and metabolism cycles in the body and its bioavailability was poor. After being enriched by bacteria, inorganic Zn existed in the digestive tract in an organic form, combined with cellular structures [11]. They were slowly released and absorbed, following bacterial death in the intestine, so that the body could maintain higher Zn levels for an extended period of time than when supplemented with inorganic Zn.

Species diversity and abundance of the gut microbiota in rat pups were examined using 16S rRNA sequencing. The results showed that the diet was the main contributor to the gut microbiota profile, followed by the litter effect. The impact of Zn supplementation alone on the overall composition and diversity of the gut microbiota was minor, as indicated by the lack of substantial changes in gut microbiota species after ZnO therapy. Accordingly, *B. longum* CCFM1195 increased the microbial abundance and diversity within the samples, except for the high dose of Zn-enriched CCFM1195. The LEfSe analysis revealed that *Lactobacillus* and *Blautia* dominated the microbiota of the control group at the genus level, at 24.63% and 21.32%, respectively. In the model group, *Blautia*, *Ruminococcus gauvreauii*, *Flavonifractor*, and *Negativibacillus* were decreased, while the *Lachnospiraceae* NK4A136 group had increased to become the dominant genus. *Blautia* had a special anti-inflammatory effect, and the decrease in its abundance may have led to the loss of anti-inflammatory effects [55]. During Zn treatment, *Blautia* was found to be the dominant genus. With the administration of the Zn dose, the proportion of *Lactobacillus* had increased, while *Blautia* had decreased. Zn supplementation was helpful to maintain the stability and diversity of the intestinal microflora. The intestinal microbiota of *Firmicutes* had increased, especially *Lactobacillus* [56]. Moreover, the two genera were strongly out of proportion in the ZnO groups. In the CCFM1195 + ZnO-H group, the ratio of these two genera was nearly re-balanced. In the case of medium and high doses of Zn-enriched CCFM1195, the relative abundance of *Lactobacillus* exceeded *Blautia*, representing the dominant genus. Probiotics, such as *Lactobacillus*, *Bifidobacterium*, and *Enterococcus,* can improve the host’s gut microbiome balance and help restore or maintain a beneficial gut microbiome to prevent digestive disorders and potentially help growth and development [57]. Although Zn supplementation is evidently associated with specific changes in the gut microbiota, the relationship between the dynamic changes of the microbiota and Zn supplementation is not entirely established. Further research is required to evaluate the role of the gut microbiota in the process of Zn deficiency supplementation.

## 5. Conclusions

In summary, our results show that Zn deficiency has a negative effect on the growth and reproductive development of rat pups. Different Zn supplements are associated with different recovery capabilities, for various physical indicators of Zn deficiency, due to differences in absorption and utilization. The bioavailability of Zn is highest for Zn-enriched CCFM1195, less for CCFM1195 + ZnO, and least for ZnO, especially at a low dose. Therefore, the same is true for the ability to recover the body’s indicators. Great differences were observed in the microbiome in the model and Zn treatment groups. Although Zn supplementation alone did not seem to have a significant effect on microbial abundance and diversity, supplementation with *B. longum* CCFM1195 significantly increased the diversity of the intestinal microbiota and played a key role in regulating the *Lactobacillus* and *Blautia* genus balance.

## Figures and Tables

**Table 1:** Viable bacteria and Zn content of CCFM1195 powder.

Strain	Number of Viable Bacteria (cfu/g)	Zn Content (mg/g)
CCFM1195	(3.8 ± 0.3) × 10^8^	—
Zn-enriched CCFM1195	(1 ± 0.1) × 10^9^	1.7 ± 0.22

Data are presented as mean ± SEM (*n* = 5) for each parameter.

**Table 2:** Zn content in CCFM1195 powder + ZnO solution.

CCFM1195 + ZnO	H_2_O	EDTA	Digest	Sum
Zn content (μg/g)	15.91 ± 0.18	11.99 ± 0.57	1.29 ± 0.05	29.19 ± 0.51
Percent (%)	54.51	41.08	4.40	100

Data are presented as mean ± SEM (*n* = 5) for each parameter.

**Table 3:** Zn content in Zn-enriched CCFM1195 powder.

CCFM1195	H_2_O	EDTA	Digest	Sum
Zn content (μg/g)	119.94 ± 1.96	628.68 ± 3.37	2644.54 ± 4.83	3393.16 ± 10.03
Percent (%)	3.53	18.53	77.94	100

Data are presented as mean ± SEM (*n* = 5) for each parameter.

## Data Availability

All data presented in this study are available in the main body of the manuscript.

## Appendix A

**Table A1:** Information on the strains used in this experiment.

Strain Preservation Number	Latin Name
N9	*Lactobacillus plantarum*
ST-III	*L. plantarum*
N13	*L. plantarum*
CCFM8610	*L. plantarum*
CCFM8661	*L. plantarum*
X1	*L. plantarum*
CCFM242	*L. plantarum*
N8	*L. plantarum*
CCFM259	*L. plantarum*
NFM11	*L. plantarum*
138-1	*Lactobacillus reuteri*
9-5	*L. reuteri*
7-2	*L. reuteri*
AA3-8	*L. reuteri*
5-1	*L. reuteri*
CCFM339	*L. reuteri*
CCFM325	*L. reuteri*
P8-4	*L. reuteri*
9-2	*L. reuteri*
CCFM311	*L. reuteri*
CCFM1195	*Bifidobacterium longum*
FHeNJZ44M8	*B. longum*
CCFM688	*B. longum*
CCFM 752	*B. longum*
CCFM 1029	*B. longum*
FZJHZD13M2	*B. longum*
CCFM 751	*B. longum*
L6	*B. longum*
CCFM 760	*B. longum*
CCFM 689	*B. longum*
JS-WX-25-M8	*Bifidobacterium breve*
AH-WH-9-M-5	*B. breve*
FBJ-CP-1-M6	*B. breve*
CCFM683	*B. breve*
F-JS-ZJ-1-M5	*B. breve*
FSHMX3M8	*B. breve*
FBJHD5M2	*B. breve*
JS-WX-23-M8	*B. breve*
FSH-MX-3M1	*B. breve*
CCFM1026	*B. breve*

**Table A2:** Culture medium components of mMRS.

Components	Volume(1 L)
Trypyone	10 g
Beef extract	10 g
Yeast extract	5 g
D(+)-Glucose anhydrous	20 g
Sodium acetate	2 g
MgSO_4_·7H_2_O	0.1 g
MnSO_4_·H_2_O	0.05 g
Diammonium hydrogen citrate	2 g
K_2_HPO_4_	2 g
Tween 80	1 mL
L-Cysteine	1 g

**Table A3:** Polymerase chain reaction (PCR) reaction system.

PCR System (50 μL)	
2 × Taq MasterMix	25 μL
ddH_2_O	20 μL
341F	1.5 μL
806R	1.5 μL
DNA template	2 μL
